# Thrombosis Associated With Cytomegalovirus (CMV) in a Kidney Transplant Recipient

**DOI:** 10.7759/cureus.17745

**Published:** 2021-09-05

**Authors:** Lin Wang, Young Hsu, Neeraj Sharma

**Affiliations:** 1 Nephrology, University of Southern California, Los Angeles, USA; 2 Internal Medicine, Keck School of Medicine, Los Angeles, USA

**Keywords:** acute cmv infection, cytomegalovirus, venous thromboembolism, kidney transplant, deep vein thrombosis (dvt)

## Abstract

Cytomegalovirus (CMV) infection is a common infectious complication after kidney transplantation. Indirect effects of CMV infection include an increased risk of secondary infections, increased risk of acute rejection, and chronic allograft dysfunction. However, it is not well known that CMV may also increase the risk of venous and arterial thrombosis. Here, we present a case of acute deep venous thromboembolism associated with acute CMV disease in a kidney transplant recipient. We also performed a literature review of cytomegalovirus-associated thrombosis in immunocompromised individuals.

## Introduction

Thrombosis remains a significant complication after kidney transplantation [1]. Evidence suggests kidney transplant recipients manifest features of a chronic hypercoagulable and persistent inflammatory state, with alterations in fibrinogen and factor VII coagulant activity [2].

In addition, this group of patients not only suffer from both inherited and acquired risk factors for thrombosis, but also conditions that are related to transplantation such as delayed graft function, history of a prior thrombosis, immunosuppressive drugs, post-transplant erythrocytosis, proteinuria, and infectious causes [3]. It has been postulated that certain infectious pathogens may influence a thrombotic disorder. Cytomegalovirus (CMV) specifically has been suggested to play an important role in unprovoked thrombosis [4].

CMV infection following kidney transplantation is associated with poor patient and graft survival [5]. Transplant patients who are at the highest risk for CMV infection are seronegative recipients of seropositive donor organs (so-called, D+/R-). Acute CMV infection in kidney transplant recipients can manifest as CMV syndrome or CMV disease [6]. Venous thromboembolism is an underestimated but significant complication of acute CMV infection. Herein, we describe a case of a tissue-invasive CMV disease associated with an acute thrombotic event in a kidney transplant recipient.

## Case presentation

Here, we present a case of a 64-year-old male with a past medical history of insulin-dependent diabetes mellitus, left upper extremity deep vein thrombosis (DVT), and end-stage renal disease who underwent a deceased donor kidney transplantation approximately 16 months prior. He initially presented to an outside hospital with a five-day history of sore throat, cough, subjective fevers, fatigue, and increasing pain in his right calf. He was empirically treated for presumed pneumonia with ceftriaxone and azithromycin. At that time, coronavirus disease 2019 testing was negative. Notably, he was found to have acute thromboses in the right posterior tibial and right popliteal veins. He was initially started on a heparin infusion and subsequently transitioned to apixaban 5 mg twice a day prior to discharge home. Three weeks later, the patient presented to our outpatient transplant clinic for follow-up. At this time, he continued to endorse sore throat and cough, with a new complaint of diarrhea for the past week. Serum CMV PCR was checked and resulted in a viral load of 35,900 IU/mL. The patient was consequently admitted for CMV viremia and concern for tissue-invasive disease.

Pertinent renal transplant history includes that the donor was CMV positive and the recipient was CMV negative (D+/R-). He had completed a year of CMV prophylaxis with valganciclovir post-transplant, with his last dose about 2.5 months before his admission for CMV viremia. His home immunosuppression regimen consisted of tacrolimus 1.5 mg daily, mycophenolate mofetil 500 mg twice a day, and prednisone 5 mg daily. In addition, the patient denied a history of smoking, recent surgery, trauma, or obesity. 

**Figure 1:**
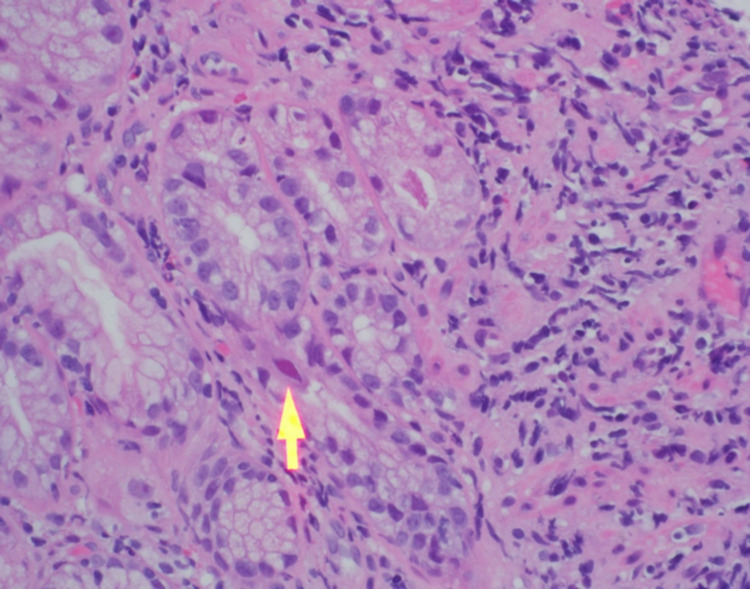
Hematoxylin and eosin staining at 40x for CMV. A CMV-infected cell is indicated by the yellow arrow. The cell infected with CMV is larger with granular intracytoplasmic inclusions giving the so-called “Owl’s Eye” appearance. CMV - Cytomegalovirus

During the hospitalization, the patient was started on ganciclovir given the aforementioned concern for tissue-invasive disease and viremia. Complete blood count, comprehensive metabolic panel, urinalysis, immunoglobulin levels, and tacrolimus trough were unremarkable. CT chest with contrast showed non-specific right pulmonary micronodules without evidence of pneumonia. Tacrolimus was resumed, however at an adjusted dose to maintain a lower trough goal. Mycophenolate mofetil was held given the status of CMV reactivation. Prednisone was resumed at his regular home dose of 5 mg daily. The patient underwent esophagogastroduodenoscopy (EGD) and colonoscopy with biopsies that stained positive for CMV in the lower esophagus consistent with CMV esophagitis (Figure 1). Ophthalmology was consulted and performed a dilated fundus examination without evidence of CMV retinitis.

Four days after starting ganciclovir, CMV PCR viral load had decreased to 1,070 IU/ml. On hospital day 7, the patient’s diarrhea resolved so ganciclovir was switched to valganciclovir 900 mg twice a day. Subsequent weekly checks of CMV PCR viral load were 120 IU/mL, then undetectable. The patient was discharged home with valganciclovir.

On outpatient follow-up, his serum CMV PCR had been undetectable on multiple occasions thus valganciclovir was discontinued after the completion of six weeks of treatment following his hospitalization. Repeat EGD two months after discharge demonstrated clearance of CMV disease in the esophagus. Additionally, the patient had been referred to hematology for further evaluation of right lower extremity DVT. Per hematology, the patient did not demonstrate any strong provoking factors for thromboembolism on history aside from his recent acute CMV infection.

This case raises the question if our transplant recipients with CMV are at higher risk for thromboembolic events and how long should the duration of anticoagulation be.

## Discussion

CMV (human herpes virus-5) is a widespread virus belonging to the herpes virus family that establishes latent infection following primary infection. Patients who are CMV seropositive have a latent infection and the risk of CMV reactivation is highest in the setting of systemic immunosuppression. Approximately 60% to 80% of donors and recipients are latent CMV seropositive with a risk of de novo disease reactivation after transplantation [5]. Despite prophylaxis, CMV viremia is reported to occur in approximately one-third of patients, the majority of who are often D+/R- recipients including those treated for rejection [7].

CMV infections remain an important cause of morbidity, mortality, and cost in transplantation. Infections may present with a wide array of syndromes ranging from meningoencephalitis to enteritis/colitis and hepatitis. In addition, CMV infection has been associated with thromboembolic events. Latent CMV has been found on endothelial cells in both venous and arterial vascular walls. It has been postulated that CMV-induced endothelial injury results in a prothrombotic state by several mechanisms [8]. Endothelial cells infected with CMV have shown increased tissue factor expression, which plays a key role in hemostasis and thrombosis formation [9,10]. Prior literature has also demonstrated that some enveloped viruses, including CMV, contain phosphatidylserine-like procoagulant activity on their surface which predisposes to thrombus formation [9,11].

Kidney transplant recipients show a high prevalence of thrombotic events compared with the general population. In 2004, Irish et al. mention that early allograft thrombosis had been reported in between 2% and 8% of renal transplants [12]. Several risk factors for renal allograft thrombosis have been reported in the literature, including but not limited to: prior history of thrombosis, diabetes mellitus, systemic lupus erythematosus, protein C or S deficiency, factor V Leiden, lupus anticoagulant activity, anticardiolipin antibodies, MTHFR C676T gene mutation, antibodies against b2-glycoprotein-1, multiple vessels on allograft, re-transplantation, use of continuous ambulatory peritoneal dialysis, and use of cyclosporine or anti-CD3 monoclonal antibody therapy [13-16]. Screening for the aforementioned risk factors is crucial in the workup and evaluation of a renal transplant recipient who develops acute renal allograft thrombosis, particularly in the setting of CMV infection.

A case-control study compared the frequency of thrombosis in a heterogeneous group of patients with confirmed acute CMV infection and a matched control group in whom CMV infection was excluded. The incidence of thrombosis was significantly higher in patients with acute CMV infection (6.4%) than in uninfected controls (0%) [17]. Furthermore, in 2004, Kazory et al. reported several cases of nonhospitalized renal transplant recipients with no special predisposing risk factors, which developed venous thrombi following an acute CMV infection. Over 85% of those patients had never had a thromboembolic event in the past [9]. This further suggests that CMV infections are likely implicated in venous thrombus formation. The more recent retrospective study conducted within the OSF HealthCare System in Peoria, IL divided studies groups into active CMV infection, seropositive immunoglobulin G (IgG) without active infection, and seronegative IgG and immunoglobulin M. The study showed 10% of the patients were diagnosed with DVT within one year of acute CMV diagnosis compared to 3.1% in the seropositive group and 3.0% in the seronegative group [18]. This suggested that acute CMV infection could be a reversible risk factor of DVT. Recurrence of venous thrombosis has also been associated with CMV infection. In a retrospective analysis conducted by Lijfering et al., the 10-year recurrence rate of venous thrombosis following renal transplantation in CMV seronegative individuals was calculated to be around 10%. Meanwhile, in seroconverted and seropositive recipients the recurrence rate of thrombosis was as high as 51% and 59%, respectively [19].

## Conclusions

In conclusion, acute CMV infection should be considered a risk factor for venous thromboembolism. Diagnosis of acute CMV infection in patients with acute thrombosis should redefine the thrombotic event as provoked rather than unprovoked; therefore, limiting the duration of anticoagulation treatment.
